# Supplementary material for: Demystifying Chronic Kidney Disease of Unknown Etiology (CKDu): Computational Interaction Analysis of Pesticides and Metabolites with Vital Renal Enzymes
Source: Biomolecules. 2021 Feb 10;11(2):261. doi: 10.3390/biom11020261 (PMC7916818; doi:10.3390/biom11020261)
Supplement: Supplementary file 1 [file biomolecules-11-00261-s001.pdf]

Supplemental information for

Demystifying Chronic Kidney Disease of Unknown etiology (CKDu): Computational Study of Pesticides and Their Metabolites as Possible Risk Factors

R.M.H. Rajapaksha[1], D.R. Pandithavidana[1], Jayangika N. Dahanayake[1*]

[1]Depertment of Chemistry, Faculty of Science, University of Kelaniya, Dalugama, 11300, Western Province, Sri Lanka.

[*]Corresponding author: Jayangika N. Dahanayake

Address: Jayangika N. Dahanayake, Senior Lecturer, Department of chemistry, Faculty of Science, University of Kelaniya, Dalugama, 11300, Sri Lanka.

Email: Jayangika N. Dahanayake

Table S1: Full names of the pesticides and metabolites.

Abbreviation	Full name of the chemical species
AC	Acetphate
MED	Methamidophos
CP	Chlorpyrifos
TCP	3,5,6- trichloro-2-pyridinol
DEP	Diethyl phosphate
DEPT	Diethyl thiophosphate
DZ	Diazinon
IMP	2-isopropyl-6-methyl-4(1H)-pyrimidinone
FN	Fenthion
FX	Fenoxon
FNS	Fenthion sulfone
FXS	Fenoxon sulfone
FXSX	Fenoxon sulfoxide
FM	Fenamiphos
FMS	Fenamiphos sulfone
DFS	Desisopropyl fenamiphos sulfone
FMSX	Fenamiphos sulfoxide
DFSX	Desisopropyl fenamiphos sulfoxide
PH	Phenthoate
PC	Phenthoate acid
DEMP	Desmethyalphenthoate
DEMPA	Desmethyalphenthoate acid
DEMPO	Desmethyalphenthoate oxon
DEMPOA	Desmethyalphenthoate oxon acid
DMTP	Dimethyl thiophosphate
IM	Imidachloprid
6-CIPHD	1-(6-chloro-3-pyridylmethyl)-2-(hydrox-yimino)-3,4-didehydroimidalozolidene
IG	Imidachloprid guanidine
6-CINA	6-Chloronicotinic acid
PF	Profenofos
M1	Des-S-propylated profenofos
M3	Despropylated profenofos
M4	Desethylate profenofos
BCP	4-bromo-2-chlorophenol
DM	Dimethoate
DMP	Dimethyl monophosphate
MMP	Monomethyl phosphate
GP	Glyphosate
AMPA	Aminomethylphosphonic acid
QP	Quinalphos
HQ	Hydroxyquinoline

Table S2: Ramachandran map of models calculated with PROCHEK and results of ERRAT, VERIFY 3-D and ProSA test.

**Model Description**		**GLS**	**ASK 1**	**GST**	**CP 450**	**PKC**	**AChE**	**AMPK**
**Ramachandran plot**	Residues in most favored regions [A,B,L]	238/ 87.7%	415/ 93.3%	666/ 93.0%	727/ 90.6%	515/ 90.4%	792/ 90.7%	680/ 93.0%
	Residue in additional allowed regions [a,bl,p]	30/ 11.2%	30/ 6.7%	41/ 5.7%	71/ 8.8%	50/ 8.8%	78/ 8.9 %	45/ 6.2%
	Residues in generously allowed regions [~a,~b,~l,~p]	3/1.1%	0/0.0%	8/1.1%	2/0.3%	5/ 0.8%	3/0.4%	6/0.8%
	Residues in disallowed regions	0/0.0%	0/0.0%	1/0.1%	2/0.3%	0/ 0.0%	0/ 0.0%	0.0/ 0/0%
	Number of non-glycine and non-proline residues	268/ 100.0%	445/ 100.0%	716/ 100.0%	802/ 100.0%	570/ 100.0%	873/100.0 %	731/ 100.0%
	Number of end-residues (excl. Gly and Pro)	3	12	4	12	6	7	6
	Number of Glycine residues	28	48	72	46	52	105	85
	Number of Proline residues	15	26	44	68	36	96	40
	Total number of residues	314	531	836	928	664	1081	862
**ERRAT overall quality score**		95.74	96.20	99.00	88.22	94.667	92.14	90.12
**VERIFY 3-D (3D-1D score >0.2)**		100.00%	88.19%	98.81%	83.46%	84.50%	74.55%	85.40%
**ProSA Z-score**		-8.95	-6.38	-7.65	-9.84	-7.36	-9.93	-7.93

Table S3: Autodock 4 grid parameters

Protein	Grid center coordinates in XYZ	Number of grid points in XYZ	Grid spacing (Å)
GLS	15.162, -7.291, -23.081	126 X 126 X 126	0.375
ASK 1	8.471, 30.394, 12.372	126 X 126 X 126	0.600
GST	48.569, 5.28, 49.709	126 X 102 X 126	0.497
CP 450	31.864, -21.827, 28.81	126 X 126 X 126	0.519
PKC	36.799, 48.555, 32.431	104 X 126 X 120	0.525
AChE	-1.859, 64.976, 352.368	126 X 126 X 126	0.547
AMPK	-33.123, 46.079, -7.256	126 X 112 X 126	0.780

Table S4: Predicted amino acid residues of the active sites found by GASS-WEB server.

Protein	Amino acid residues of the active site
**AChE**	SER 200 A; TRP84 A; TYR334 A; PHE331 A; TYR121 A
	SER 467 A;LEU 239 B;HIS 255 B;LEU 163 C;TYR 257 B
	LEU 278 A;SER 127 A;GLN 123 A;ASP 130 A;HIS 75 A
**AMPK**	GLY 121 B;SER 203 B;ALA 204 B;HIS 447 B;GLU 334 B
	GLY 120 B;SER 203 B;ALA 204 B;HIS 447 B;GLU 334 B
	GLY 122 B;SER 203 B;ALA 204 B;HIS 447 B;GLU 334 B
**ASK 1**	SER 821 B;GLY 689 B;ASP 803 B;THR 838 B;ARG 767 B
	GLY 824 B;SER 826 B;TYR 691 B;ASP 863 B;HIS 801 B
	TYR 793 B;HIS 734 B;LYS 709 B;ASP 822 B;HIS 729 B
**CP 450**	THR 252 A; VAL 247 A; VAL 396 A; THR 185 A;LEU 244
	CYS 357 A;ALA 370 A;ARG 365 A;THR 252 A;ASP 251 A,
	SER 437 B;ARG 446 A;ASP 425 B;GLY 444 A;ARG 130 A
**GLS**	SER 286 A;GLU 381 A;ASN 388 A;VAL 484 A; TYR 249 A
	MET 508 A;SER 482 A;ASP 506 A;GLU 381 A;LYS 481 A
	LYS 292 A;TYR 414 A;ILE 391 A;GLY 337 A;LYS 399 A
**GST**	SER 165 A;ASP 98 B;GL 64 A;TRP 34 A;GLN 51 A
	SER 149 C;GLY 73 C;ASP 90 C;THR 67 D;ARG 100 C
	SER 149 C;GLY 73 C;ASP 90 C;THR 67 D;ARG 13 C
**PKC**	PHE 659 A;SER 408 A;MET 420 A;ASP 341 A;HIS 407 A
	LYS 481 B;TYR 507 B;ILE 462 B;GLY 453 B;LYS 460 A
	PHE 661 A;SER 408 A;MET 420 A;ASP 341 A;HIS 407 A
**PKD 2**	NONE

Table S5: The water box size and number of water molecules in each solvent box of protein ligand complexes.

Protein-ligand complex	Size of the Solvent box	Number of Water molecules
Ache-FM	9.70260 x 9.70260 x 9.70260	28311
Ache-FMS	9.70079 x 9.70079 x 9.70079	28303
ASK1-FM	8.10273 x 8.10273 x 8.10273	16688
ASK1-FMS	8.10464 x 8.10464 x 8.10464	16694
PKC-IM	9.20054 x 9.20054 x 9.20054	24693
PKC-6CIPHD	9.20086 x 9.20086 x 9.20086	24694
GST-IM	8.48899 x 8.48899 x 8.48899	18679
GST-6CIPHD	8.49111 x 8.49111 x 8.49111	18681
AMPK-FN	14.87117 x 14.87117 x 14.87117	102790
AMPK-FNS	14.68086 x 14.68086 x 14.38093	102891

Table S6: Templates found by BLAST search that have ≥80% similarity to the query sequence.

Protein	BLAST Results					
	**Max Score**	**Total Score**	**Query Cover**	**E Value**	**Precent Identity**	**Accession**
AMPK	1001	1001	100%	0.00	100.00%	6C9H
	998	998	100%	0.00	99.79%	6C9F
	968	968	98%	0.00	97.30%	4QFG
	962	962	96%	0.00	99.35%	4REW
	960	960	96%	0.00	99.14%	4RER
	956	956	97%	0.00	94.89%	4CFH
PKC	725	725	100%	0.00	98.30%	3PFQ
	617	617	97%	0.00	84.26%	31W4
	614	614	97%	0.00	83.97%	4RA4
ASK 1	561	561	100%	0.00	100.00%	2CLQ
	560	560	100%	0.00	100.00%	5VIL
	559	559	100%	0.00	100.00%	5UOX
	559	559	100%	0.00	100.00%	5OUR
	560	560	100%	0.00	99.63%	4BIB
AChE	1100	1100	100%	0.00	100%	4BDT
	1100	1100	100%	0.00	100%	4PQE
	1100	1100	100%	0.00	100%	4EY4
	1099	1099	100%	0.00	100%	6O4W
	1095	1095	99%	0.00	100%	3LII
	1093	1093	99%	0.00	100%	1B41
	1099	1099	100%	0.00	99.82%	1F8U
CP 450	1003	1003	100%	0.00	100.00%	1W0E
	1001	1001	99%	0.00	100.00%	4NY4
	1000	1000	99%	0.00	100.00%	3TJS
	100	100	99%	0.00	100.00%	1TQN
	998	998	99%	0.00	99.79%	6MA7

Table S7: Amino acids responsible for ligand protein interactions and the types of dominate interactions by each amino acid. Blue –hydrogen bonding, yellow – salt bridges, red – π-cation interaction, green – halogen bonding and, magenta - π-π stacking.

Pesticide		Protein						
		AMPK	PKC	GLS	ASK 1	AChE	GST	CP450
AC	AC	MET84C, ARG117C, PRO127C, VAL129C	THR504A, ARG465A, ILE512A, ALA509A, VAL522A	PHE322A, LEU323A, TYR394A	ALA929B, HIS795B, ILE864B, LYS860B	TYR341A, PHE338A, TRP286A, ARG296A, PHE295A	TYR49C, ASP98D, LYS103D, GLN125D	LYS34A, PHE33A, TYR75A
	MED	GLY16A, ASP117B, HIS17A,	CYS502A, ALA509A	LYS399A, CYS400A, PHE401A	ARG828B, LYS827B, GLU724B	SER293A, TRP286A, ARG296A	GLN64C, GLY95C, GLN125D, LYS102D	LYS34A, PHE33A, TYR75A
CP	CP	ARG69C, ALA70C, ARG151C, TYR164C, ILE165C, PRO225B	ALA652C, VAL623C, LEU258D, TRP455D, GLN622C	TYR466A, ARG387A, LYS320A, ASN335A,	LYS912B, GLU905B, GLU902B, ILE903B	SER125A, TYR124A, GLU202A	ARG74D, TYR79D, ALA86D, ARG74C, GLN83C	PHE302B, GLY444B, ILE443B
	TCP	LEU24A, GLU96A, VAL98A, LEU148A	ASN663A, LEU406A, GLU665A, LEU394A, LYS399A	SER314A, LYS320A, VAL334A, PHE318A, HIS330A, SER314A	SER314A, LYS320A, VAL334A, PHE318A, HIS330A	LYS53B, PHE37B	GLN5A, PRO53A, SER65A, GLN64A	GLY480B, CYS580B, MET59B, LYS55B, LEU477B

	**DEP**	ASN66C, LYS46C, LEU228B, LEU215B, LYS395A	ASN663A, LEU406A, GLN405A, LYS399A	LYS320A, ASN335A, ALA336A, ILE391A, ARG387A, PHE322A	LEU728B, GLU724B, LYS827B, ARG828B	SER196B, LYS23A, ARG21A, ARG485B, ARG224B	ARG74C, TYR79D, ARG74D, ALA86C	ILE443B, ARG130B, TRP126B, ARG105B, ARG440B, ILE118B
	**DETP**	ARG117C, VAL82C, LEU128C, MET84C	PHE661A, LEU406A, GLN405A, LYS399A, ASN663A	CYS400A, MET407A, LYS396A, PHE401A	ILE903B, GLU902B	ARG296A, TRP286A, PHE295A	TYR49A, GLN51A, PRO53A, GLN64A, TYR63A, SER65A	LEU479B, LYS55B, MET59B, CYS58B, PHE57B
**DZ**	**DZ**	ARG117C, MET84C, VAL129C, ILE149C, ARG151C	ASN663A, LEU667A, PRO401A, LYS399A, GLU465A	LYS320A, ARG387A, ILE391A, ASN335A, VAL334A, ALA336A	LYS912B, ILE903B, GLU902B, LEU916B	TYR124B, SER125B, TRP86B, TYR337B	LYS44B, GLN125A, TYR49B, PRO128A, THR131A	ARG372A, MET137A, PHE57A, LEU482A, LEU483A, ILE369A, THR309A, PHE304A, GLU308A

	**IMP**	ARG117C, VAL82C, LEU128C, MET84C	LEU467A, TYR507A, ALA526A, ILE508A, VAL522A, ASP523A, ILE512A, ARG465A	LYS320A, VAL334A, ARG387A, HIS330A, ASN335A, PHE322A, ILE391A, ALA336A	GLU755B, GLN756B, VAL757B, LEU810B, VAL694B, LEU686B	LYS53A, TRP56A, PRO52A, GLU185A, ASN186A, VAL12A, TRP182A, GLY14A, LEU178A	GLN64D, PRO53D, GLN51D	GLY109A, ARG150A, PHE108A, GLU122A, LYS115A, ILE120A, VAL111A
	**DEP**	ASN66C, LYS46C, LEU228B, LEU215B, LYS395A	ASN663A, LEU406A, GLN405A, LYS399A	LYS320A, ASN335A, ALA336A, ILE391A, ARG387A, PHE322A	LEU728B, GLU724B, LYS827B, ARG828B	SER196B, LYS23A, ARG21A, ARG485B, ARG224B	ARG74C, TYR79D, ARG74D, ALA86C	ILE443B, ARG130B, TRP126B, ARG105B, ARG440B, ILE118B
	**DETP**	ARG117C, VAL82C, LEU128C, MET84C	PHE661A, LEU406A, GLN405A, LYS399A, ASN663A	CYS400A, MET407A, LYS396A, PHE401A	ILE903B, GLU902B	ARG296A, TRP286A, PHE295A	TYR49A, GLN51A, PRO53A, GLN64A, TYR63A, SER65A	LEU479B, LYS55B, MET59B, CYS58B, PHE57B
**DM**	**DM**	ARG117C, MET84C	LEU467A, THR504A	LEU321A, LYS320A, ARG387A	ARG705B, GLY759B, GLN756B	TRP86A, ARG296A, TYR124A	GLN123C, LYS102C	GLY40A, TYR75A, LYS34A, PHE33A

	**MMP**	LYS46C, LEU215B, LYS295A, ASN66C	ASN663A, LEU406A, GLN405A, LYS399A, LEU396A	LYS320A, ARG387A	LYS827A, ARG828A, GLU724A	ARG247A, PHE297A, TRP236A	ARG186C, ASP23C, LYS188C	ILE118A, TRP126A, ARG130A, ASN441A, ARG440A, ARG105A
	**DMP**	ASN66C, LYS46C, LEU215B, LYS395A	ASN663A, LYS399A, GLY398A, LEU396A	LYS320A, ARG387A	GLU724B, ARG828B, LYS827B	GLY58A, ARG16A	ARG74C, ARG74D	ARG105A, ARG440A, TRP126A, ARG130A, ILE118A
**FN**	**FN**	ARG117C, MET84C ILE149C	VAL356A, VAL423A, ASP484A, THR404A	VAL334A, ALA336A, LEU321A, LYS320A	VAL757B, VAL738B, VAL694B, LYS709B, LEU810B, SER821A, ASO822B	TYR341A, TRP286A, PHE338A, ARG296A, PHE295A, TYR124A, PHE297A, TYR337A,	PRO128D, GLN125D, LYS102D, ASP98C	PRO485A, LYS173A, SER315A, VAL489A, LYS173A
	**FX**	ARG117C, LEU128C, MET84C, ILE149C	ASN663A, GLN405A, LYS399A, GLY398A, GLU665A	LYS320A, VAL334A, ARG387A, ASN335A, ALA336A	ALA707B, LEU686B, LEU810B, VAL694B, LYS709B, VAL738B, SER821B, ASP822B	TYR341A, TRP286A, PHE338A, ARG296A, PHE295A, TYR124A, PHE297A	ARG74C, ARG74D, TYR79C, ALA86C	PHE220A, CYS239A, PRO242A, ARG243A

	**FNS**	MET84C, VAL129C, ARG151C, HIS150C, ILE149C	ASN663A, GLN405A, LYS399A, LYS611A, LYS481A	LYS320A, ASN335A, ALA336A, ARG387A, VAL334A	VAL757B, LEU810B, SER821B, ASP822B, ALA707B, LYS709B	TYR341A, PHE338A, ARG296A, TYR337A, TYR124A, PHE295A	LEU52D, ARG13D, VAL10D, ASN204D, TYR108D	ARG130A, PHE137A, ARG446A
	**FXS**	VAL129C, MET84C, THR86C, ARG151C, HIS150C, THR88C, IEL149C	ASN663A, LEU667A, GLN405A, LYS481A, LYS611A, LYS399A,	LYS320A, LEU321A, ALA336A, ARG387A, VAL334A, ASN335A,	VAL757B, ALA707B, LYS709B, ASP822B, VAL738B, LEU810B	TYR341A, ARG296A, PHE338A, TRP337A, TYR124A, PHE195A	ARG74C, ARG74D, ALA86C, TYR79C	PHE219A, ARG212A, PRO242A, ARG243A, CYS239A, ILE238A
	**FXSX**	VAL129C, MET84C, THR86C, ARG151C, HIS150C, THR88C, ILE149C	ASN663A, LEU667A, GLN405A, LYS481A, LYS611A, LYS399A, GLN665A	LYS320A, ARG387A, ASN335A, VAL334A, ALA336A	VAL757B, LEU810B, SER821B, ASP822B, LYS709B, ALA707B	TYR341A, ASP74A, TYR337A, 1RG296A, PHE295A	PRO128D, GLN125D, LYS102D,	ARG212A, PRO242A, CYS239A, PHE220A, PHE219A

FM	**FM**	HIS150C, ARG69C, HIS297C, GLU295C, ARG298C, ILE239C, SER241C, PHE243C, LYS242C, HIS297C	VAL356A, PHE353A, ASP484A, TYR404A, LEU394, VAL423A	LYS320A, LEU321, VAL334A,	LYS320A, LEU321A, VAL334A, ALA336A	TYR72A, TYR124A, TYR337A, SER125A, ASN87A, LEU130A, TRP86A	ARG74C, TYR79D, ARG74D, ALA86C, TYR79C	MET59A, PHE57A, LEU479A, LEU477A, LYS55A, TYR39A,
	**FMS**	LEU121C, ARG117C, MET84C, TYR86C, ILE149C, HIS150C, TYR88C, ARG151	ASN663A, LEU406A, GLU609A, GLN405A, LYS399A	ASP467A, ARG387A, ALA336A, ALA334A, LYS320A	VAL757B, GLY760B, ASP807B, ALA707B, VAL694B, LYS688B	ARG296A, TYR341A, ASP74A, TYR72A, PHE338A, TYR124A, , TYR337A, PHE295A	TYR49C, LYS102D, GLY124D, LYS127D, GLN125D, PRO128D, PHE129D	ASP217A, ASP214A, ARG212A, PRO242A, CYS239A, PRO242A, ARG234A, LEU238A
	**FMSX**	PRO127C, VAL29C, ILE149C, HIS150C, ARG151C, THR286C, MET84C	ASN663A, LEU406A, LYS481A, LYS611A, PRO401A, LYS399A, LEU667A, GLN405A	ASP467A, LYS320A, VAL334A, HIS330A	VAL757B, ALA707B, LEU686B, LYS688B, GLY760B, LEU810B, VAL694B	ASP74A, TYR341A, PHE295A, PHE338A, TYR124A, TYR337A,	PHE129D, TYR49C, ASP98D, GLN125D, GLY124D, LYS102D, PRO128D	MET59A, CYS58A, LEU479A, LEU477A, PRO447A, LEU475A, TYR399A

	**DFS**	PRO127C, VAL129C, ILE149C, HIS150C, ARG151C, THR86C, MET84C	GLY450A, ARG420A, ASN423A, TYR345A, PHE445A, TYR345A, ARG417A, ARG417A GLU312B	ASP471A, ARG387A, LYS320A, ASN339A, ALA336A, VAL334A	VAL757B, ALA707B, LYS709B, VAL694B, ASP822B, GLY760B, LEU686B, LEU810B	TRP86A, TYR124A, SER203A, TYR133A, GLY120A, SER125A	TYR49C, PRO128D, LEU99D, GLN125D, GLY124D, LYS102D	PHE219A, ARG212A, PRO242A, ARG243A, CYS239A, ILE238A
	**DFSX**	ASP117B, PRO79B, ILE115B, ALA114B, SER12A, LYS14A, GLY16A, HIS17A	GLU421A, ASN424A, GLY425A, SER476A, ASP475A, VAL423A, LYS481A, GLN405A, LYS611A, PRO401A, PRO669A	ASP467A, LYS320A, VAL334A, HIS330A	ALA707B, VAL757B, GLY760B, LYS709B, VAL694B	TYR341A, ASP74A, TYR72A, TYR124A, TRP286A , LEU28A, PHE295A, SER293A, ARG296A	LYS102D, TYR63D, SER65C, TYR49C, GLN51C, GLN64C	LYS55A, LEU477A, PHE57A, CYS58A, MET59A

PH	**PH**	ARG170C, THR167C, TYR164C, ALA225B, ILE165C, ALA70C, ARG69C, VAL68C, ARG69C	ASN663A, GLN405A, LYS399A, GLY398A, GLU665A	TYR466A, LYS320A, ARG387A, VAL334A, ASN335A, ILE391A, PHE322A, ASN336A	VAL757B, LEU810B, SER821B	TYR341A, PHE338A, ASP74A, PHE295A, TYR124A, PHE297A, ARG296A,	TYR49D, TYR63D, ASN66D, SER65D, GLN51D, ARG13D	MET59A, TYR399A, PHE57A, LEU477A
	**PC**	VAL129C, LYS126C, ARG117C, VAL82C	LYS350A, LEU373A, VAL378A, VAL377A, LYS374A	LYS320A, VAL334A, PHE322A, ALA336A, ARG387A, ASN335A	GLY759B, PRO758B, ARG705B, GLN756B	GLN474A, LEU459A, GLY422A, GLY323B, PRO25A, LEU324B, GLN325B, ARG424B	PHE129C, PRO128C, GLN125C, LYS102C, TYR49D, ASP98C	ARG105A, TYP126A, ILE118A, ILE443A, ARG440A, ARG130A
	**DEMP**	ARG117C, MET84C, LEU128C, VAL129C, ILE149C	ASP494A, GLY495A, GLY519A, GLU511A, PRO580A, SER521A	PHE318A, LYS320A, VAL334A, PHE322A, ALA336A	VAL757B, GLY759B, VAL694B, LEU810, SER761B, VAL738B, ALA707B	TYR72A, TRP286A, ARG296A, PHE338A, TYR337A, TYR124A, TYR341A	TYR63D, TYR49D, LEU52D, ARG13D, SER65D, GLN51D	PHE57A, MET59A, TYR339A, LEU477A, CYS58A,

	**DEMPA**	LEU121C, LYS126C, PRO127C, VAL129C, VAL82C, ARG117C, TYR120C	LYS350A, GLY351A, LEU373A, VAL377A, VAL378A, LYS374A	LYS320A, PHE322A, ALA336A, ASN335A, ARG387A, ILE391A, VAL334A	GLY759B, PRO758B, ARG705B, GLN756B	THR486A, PRO28A, GLN140A, TYR105A, ARG143A	TRP38A, LYS44A, GLN51C, PHE8C, GLY50C, LEU52C, TRP38C	ALA370A, ARG106A, GLU374A, ARG375A, ARG105A
	**DMP**	ASN66C, LYS46C, LEU215B, LYS395A	ASN663A, LYS399A, GLY398A, LEU396A	LYS320A, ARG387A	GLU724B, ARG828B, LYS827B	GLY58A, ARG16A	ARG74C, ARG74D	ARG105A, ARG440A, TRP126A, ARG130A, ILE118A
	**DEMPOA**	ARG151C, ILE65C, TYR164C, LYS169C, ASP224B, VAL68C, ARG69	LYS350A, VAL377A, LEU373A, LYS374A, VAL378A	ASP467A, TYR466A, VAL334A, LYS320A, ASN335A, ARG387A	PRO758B, GLY759B, GLN759B, AGR705B	ARG424B, GLN140A, ARG143A, VAL139A, PRO104A , PRO28A, TYR105A	PRO128D, GLN125D, LYS102D, TYR49C, LYS44C	ARG105A, TRP126A, ILE118A, ILE301A, PHE302A ARG130A, ILE443A, ARG440A
	**DEMPO**	ARG117C, MET84C, VAL82C, VAL129C, ILE149C, THR86C	ASN663A, LEU406A, PRO401A. LYS399A, GLN405A, LEU667A, GLU665A	HIS330A, ARG387A, LYS320A, ALA336A, ASN335A, VAL334A,	ARG705B, VAL704B, PRO758B, TYR814B, GLN756B	TYR341A, PHE338A, TYR337A ,TRP286A, TYR72A, TYR124A, PHE295A, ARG296A	ARG13C, SER65C, TYR63C, LEU52C, GLN51C, TYR49C	ARG105A, PHE108A, GLY109A, ILE120A, VAL111A, LYS115A

	**DMTP**	ARG117C, VAL82C, MET84C	ASN663A, LEU406A, GLN405A, LYS399A	LYS396A, ASP406A, MET407A, VAL408A	SER866B, ILE804B, ARG802B, THR824B, GLY841B, TYR858B	TRP286A, ARG296A, PHE295A	ARG74C, ARG74D, TYR79D	MET59A, CYS58A, LYS55A
**PF**	**PF**	ARG117C, MET84C, ILE149C, LYS126C, THR86C	GLU665A, GLN405A, LEU403A, LYS399A, PRO401A	HIS330A, TYR466A, LYS320A, SER286A, ALA336A, VAL334A, ASN335A, ARG387A	VAL757B, LEU810B, ALA707B, VAL694B	ASP74A, TYR72A, TYR124A, TYR337A, TRP86A, HIS447A	GLY80D, ARG74C, TYR79C, ALA86C, ARG74D, ALA86D	ILE184B, ILE443B, ILE118B, GLY444B, PHE302B
	**M 1**	ARG117C, VAL82C, VAL129C, MET84C, ARG151C, ILE149C	GLU665A, LYS399A, LEU406A, ASN663A, PHE661A	SER314A, ASN335A, ARG387A, PHE322A, LYS320A	PRO855A, THR838A, ARG802A, LYS827A, PRO721A, PHE839A	LEU459A. GLY323B, GLY422B, ARG424B, PRO25A, LEU324B, GLN325B	ALA86B, TYR79B, ARG74B, ALA86A, ARG74A, TYR79A	ILE38A, GLY40A, LYS34A, PHE33A
	**M 3**	ARG117C, MET84C, THR86C, ILE149C	PHE661A, GLN405A, LEU403A, LYS399A, PRO397A, ASN663A, GLU665A	PRO402A, GLY348A, ILE410A, LYS354A, GLN347A, ASP406A	VAL757B, LEU810B, LYS709B, VAL694C, ALA707B, LEU686B	TRP286B, ARG296B, TYR341B	ARG74C, ALA86D, TYR79D, ARG74D, GLN83C	LYS34A, PHE33A, GLY40A, TYR68A

	**M 4**	LYS126C, PRO127C, ARG117C, VAL129C, MET84C, ILE149C, THR86C	ASN663A, LEU667A, LEU406A, GLN605A, LYS399A	TYR466A, ARG387A, LYS289A, ASN335A,	GLU724A, PHE839A, THR838A, ARG802A, LYS827A	TYR341A, TRP337A, TRP286A, ARG296A, PHE295A	ARG74C, ALA86D, TYR79D, GLY80D, ARG74D, GLN83C	PHE137B, PHE302B, GLY444B, ILE118B, LEU443B
**QP**	**QP**	PHE160A, ASP159A, ALA158A, ILE79A, LEU148A, VAL98A, TYR97A, LEU24A	ARG624A, ASN424A, TYR422A, GLU366A, TYR368A, LYS668A, PHE666A, GLU421A, LEU367A	TYR466A, ARG387A, ALA336A, ASN335A, LYS320A, VAL334A	VAL704B, ARG705B, GLN756B, GLU755B	GLU292A, VAL365A, SER293A, LEU289A, TRP286A, ARG296A, PHE297A	PRO128D, ASP98D, LEU99D, GLN125D, LYS102D	ARG130A, ILE443A, GLU444A, PHE447A, ILE184A, PHE302A, ALA305A
	**DETP**	ARG117C, VAL82C, LEU128C, MET84C	PHE661A, LEU406A, GLN405A, LYS399A, ASN663A	CYS400A, MET407A, LYS396A, PHE401A	ILE903B, GLU902B	ARG296A, TRP286A, PHE295A	TYR49A, GLN51A, PRO53A, GLN64A, TYR63A, SER65A	LEU479B, LYS55B, MET59B, CYS58B, PHE57B

	**HQ**	VAL193B, TRP426A, VAL428A	THR504A, ALA526A, VAL522A, LEU467A, TYR507A, ILE508A	ILE221A, THR267A, SER219A, ASN494A, VAL495A, MET220A, ASP269A, ALA268A	GLY759B, GLY760B, LEU810B, VAL757B, LEU686B	TYR124A, TYR72A, TRP286A, GLU285A	TYR63D, SER65D, GLN64D, GLN51D, TYR49D	ARG440A, ARG1O5A, ILE120A, GLU122A, PRO197A PHE108A
**IM**	**IM**	SER241C, ARG298C, HIS297C, LEU276C, ILE239C, ASP244C	ASN633A, LYS481A, LEU403A, LYS399A, GLU665A	ASP467A, TYR466A, VAL334A, CYS463A, ASN335A, SER286A, LYS481A	LYS878A, PRO879A, TYR845A, GLU873A, SER763A, GLY877A	GLY323B, LEU324B, GLN140A, THR24A, PRO25A, GLY26A	GLN64C, ASP98D, GLU97C, ARG13C	PHE302B, ILE301B, GLY444B, ILE443B, ARG130B, TRP126B, ARG105B, LEU443B, ARG440B,
	**IG**	ASP224B, ILE165C, LEU163C, LEU47C, VAL68C	ASN663A, LEU406A, GLN405A, LYS399A, GLY398A, GLU665A	LEU321A, PHE322A, ASP327A, LEU323	PHE937B, GLU910B, SER908B, GLU777B	GLN140A, GLY26A, THR24A, GLY26A, GLY323B, LEU324B, PRO25A	GLN64D, GLU97D, ASP98C, GLN51D	GLU125A, ALA289A, ARG128A, LEU129A, GLU294A, LEU290A, LEU132A

	**6-CIPHD**	ALA208A, LEU214A, ASP217A	LYS371A, ALA369A, GLU390A, PHE485A, ASP484A, THR404A	PHE318A, ARG387A, ALA336A, LYS320A, VAL334A	PHE318A, LYS320A, ARG387A, ALA336A, VAL334A	ASN87A, TRP86A, SER125A, GLY122A. SER203A, HIS147A, GLU202A, GLY121A	ASN66D, ARG13D, GLN51D, SER65D, ASP98D, GLN64D	ARG440B, SER119B, GLU119B, PRO107B, ARG105B
	**6-CINA**	VAL82C, MET84C, ARG117C, LYS126C, LEU128C	LYS374A, LYS350A	VAL334A, ARG387A, HIS330A, ASN335A, LYS320A, VAL334A	LYS827A, ARG828A, LEU728A, GLU724A	ARG224B, ARG21A, GLY23A, THR24A, PRO25A, GLN325B, ARG224B, SER196B, ARG485B	TRP38A, LYS44A, LEU52A, GLN15A, GLY50A	ARG130B, TRP126B, ARG105B, ARG440B, ILE443B
**GP**	**GP**	HIS188B, GLN189B LYS427A	LYS374A, LYS350A, LYS355A	LYS399A, THR405A, GLU403A, GLY404A, PRO402A	GLU724B, LYS827B, ARG828B	ARG21A, LYS23A, ASP193B	PRO1C, THR75C, TYR3C, ASP57C	ASP428B, LYS143A, ASP348A
	**AMPA**	LYS71A, VAL84A, GLU161B, VAL162B	SER342A, LYS468A, ASP466A, ASN471A, ASP484A	GLU321A, LYS311A	ASP924B, ASP922B, LYS853B	GLU396A, LYS332A, ARG525A, ASP333A	ASN66D, SER65D, ASP98D, GLN64D	GLU125A, ARG128A

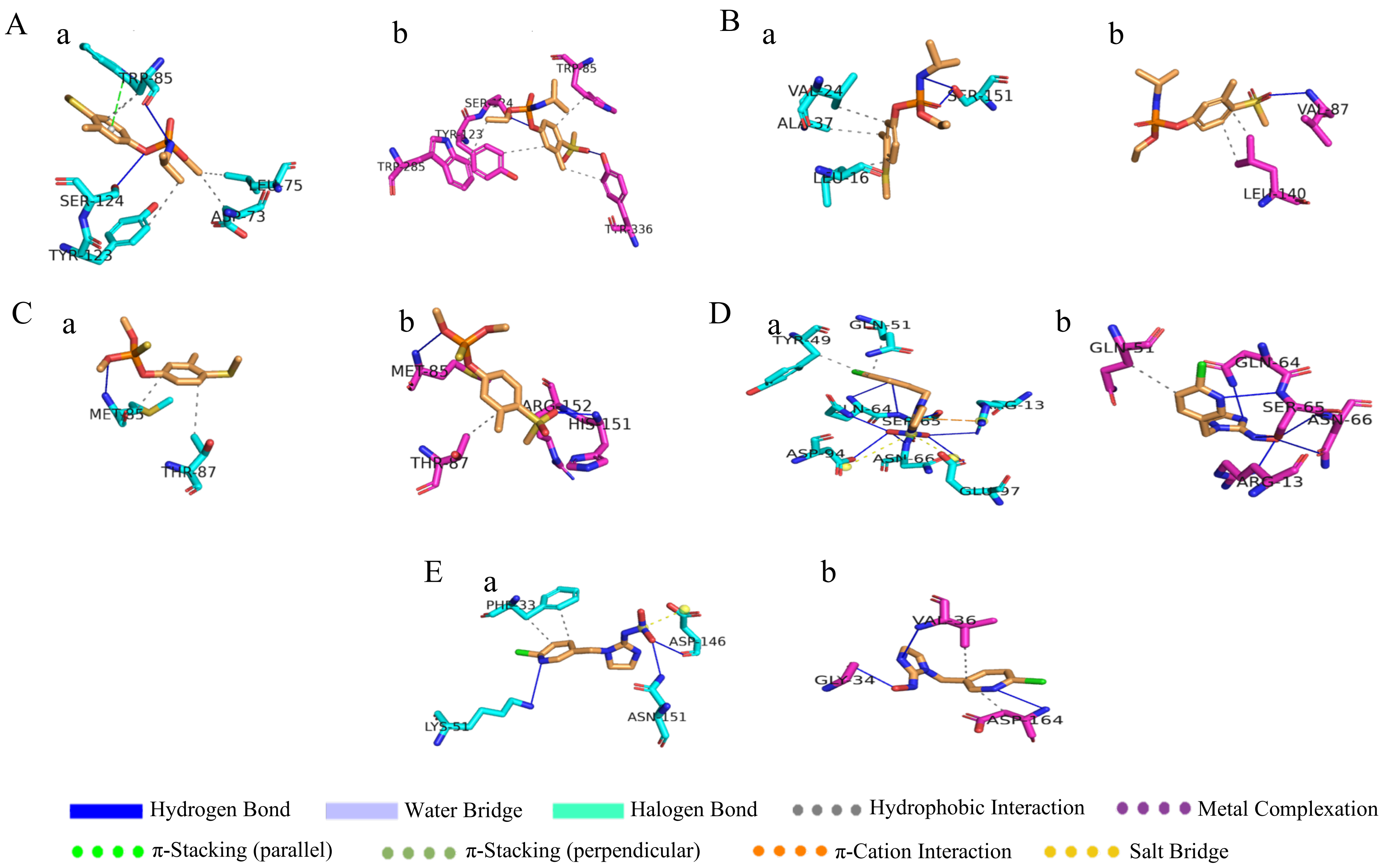

Figure S1 : Ligand -protein interactions between AChE-FM (A,a), AChE-FMS (A,b), ASK1-FM (B,a), ASK1-FMS (B,b), AMPK-Fn (C,a), AMPK-FNS (C,b), GST-IM (D,a), GST-6CIPHD (D,b), PKC-IM (E,a) and PKC-6CIPHD (E,b) complexes.

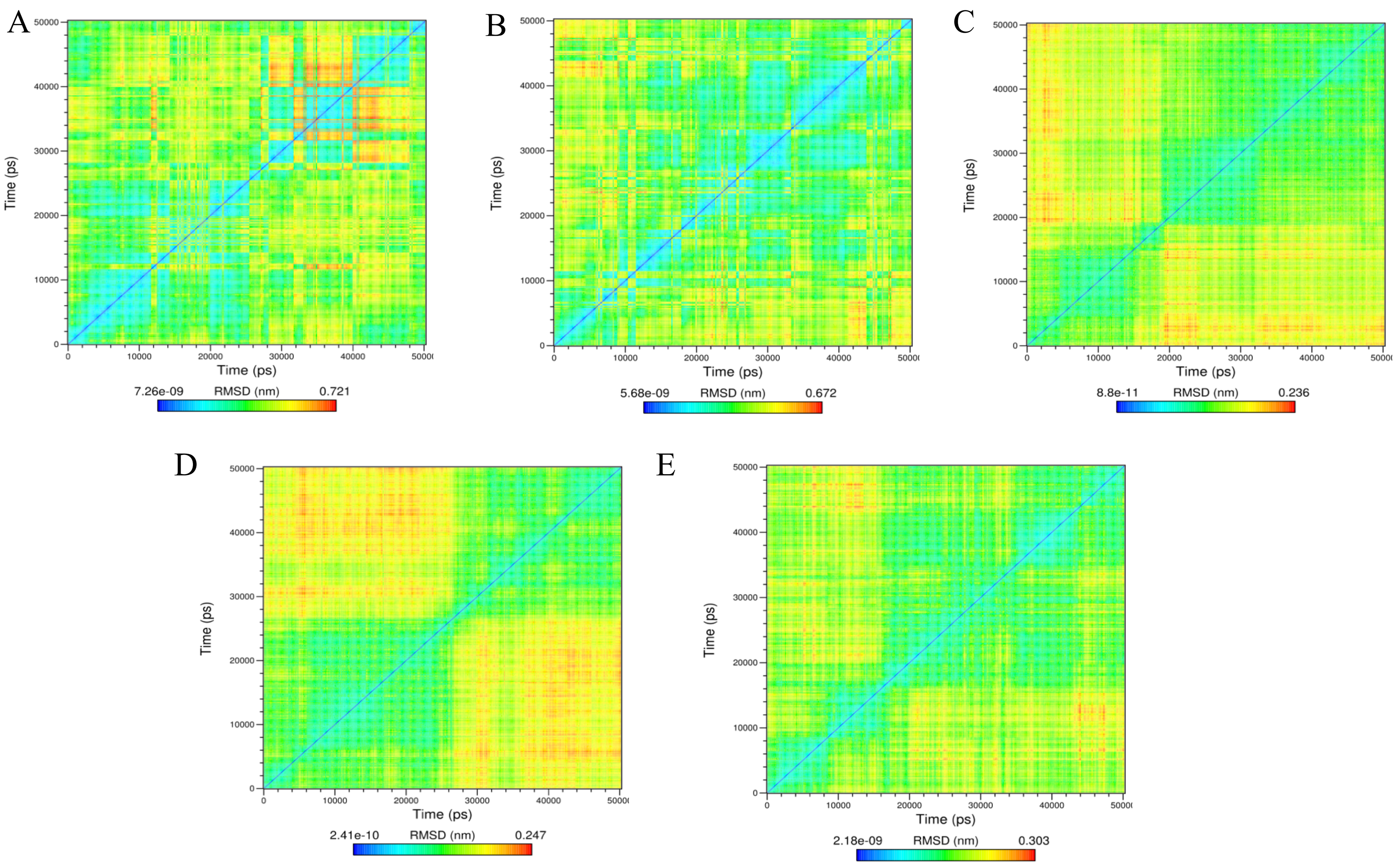

Figure S2: Backbone RMSD matrix map of A) AMPK-FN, B) AMPK-FNS, C) GST-IM, D) GST-6CIPHD, and PKC-IM complexes.

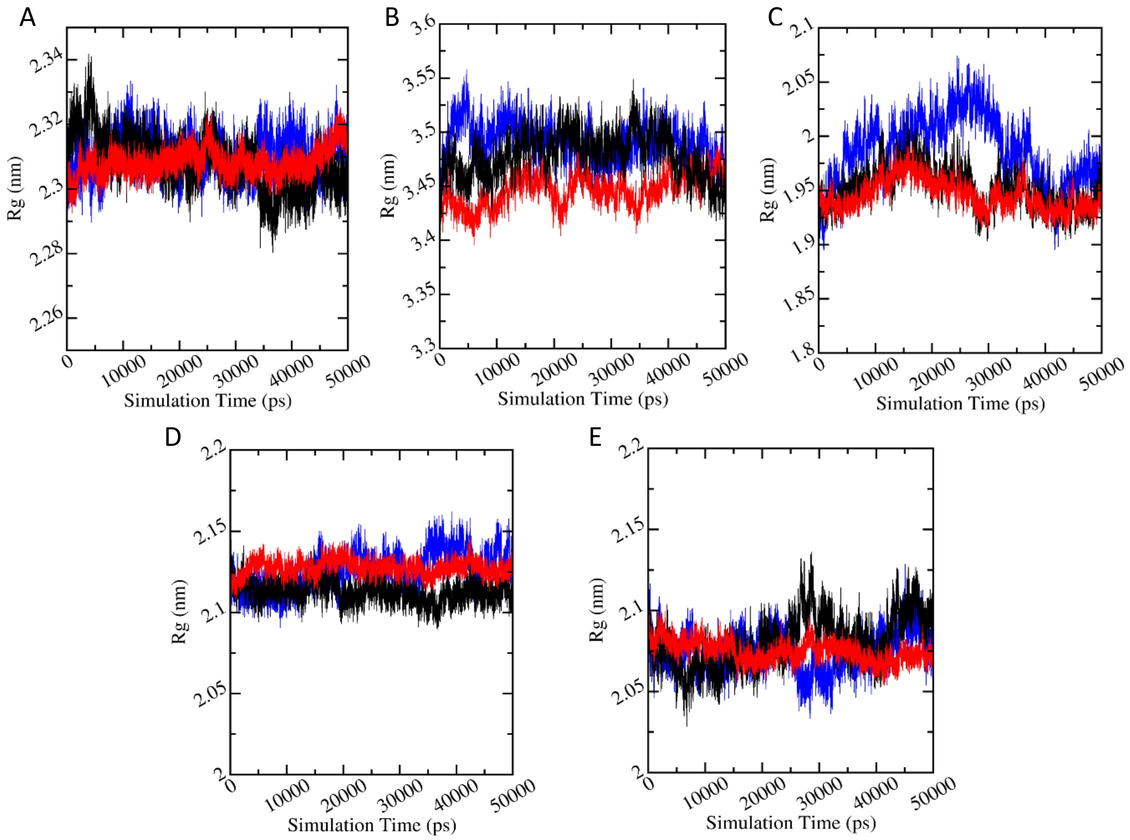

Figure S3: Plot of Radius of Gyration (nm) vs Simulation time (pS) where black, red, and blue color plots represent the parent pesticide-protein complex, metabolite-protein complex, and apo-protein, respectively. A, B, C, D, and E indicate AChE-FM/FMS AMPK-FN/FNS, ASK1-FM/FMS, GST-IM/6-CIPHD and PKC-IM/6-CIPHD complexes, respectively.

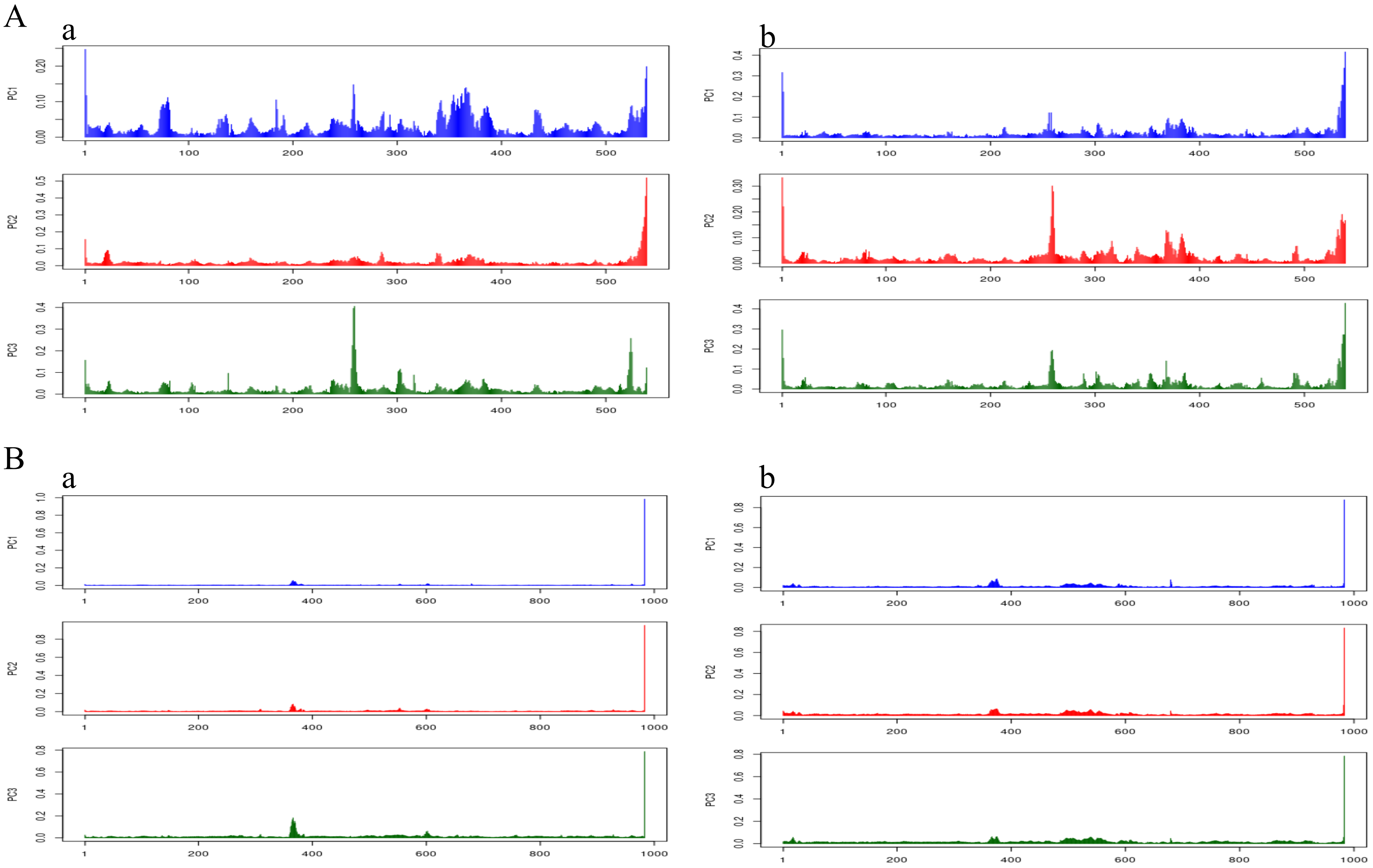
A
a
b
B
a
b
PC1
PC2
PC3
1
100
200
300
400
500
1
200
400
600
800
1000

C

a b

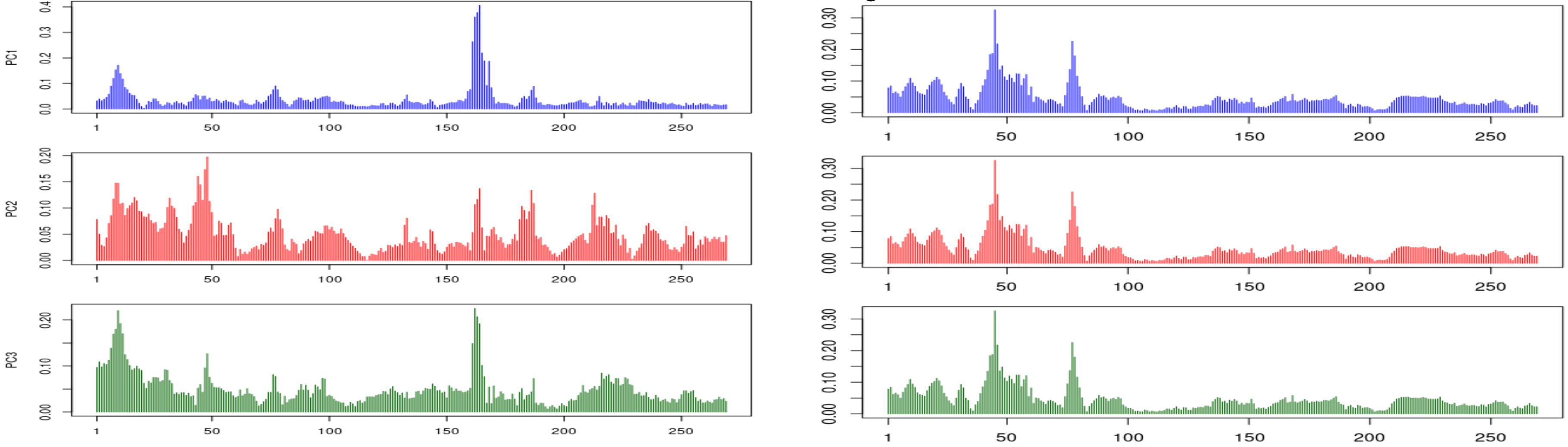

D

a b

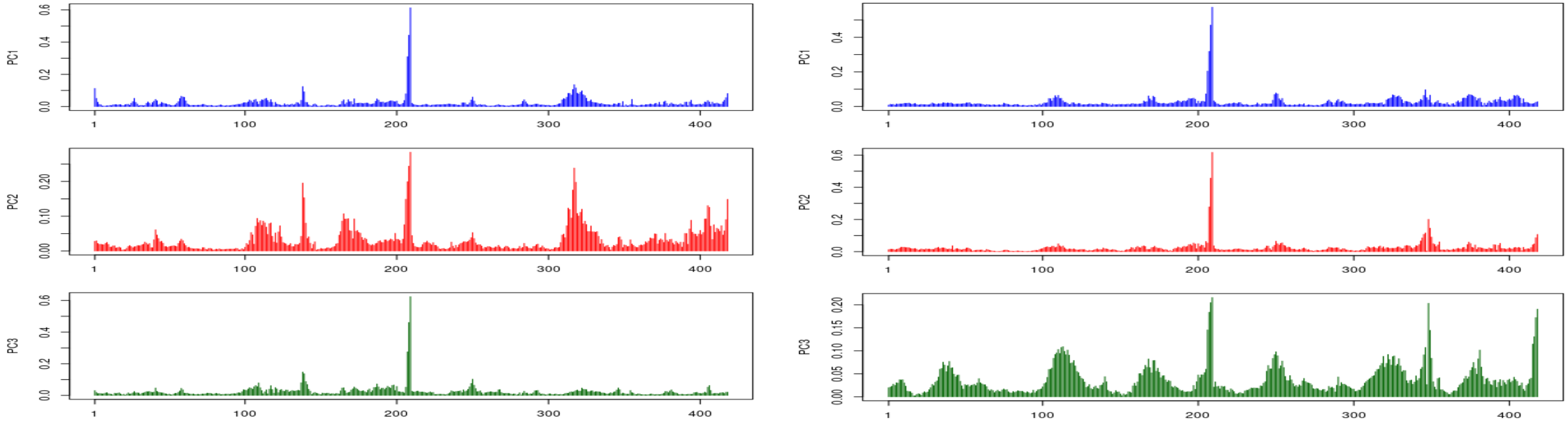

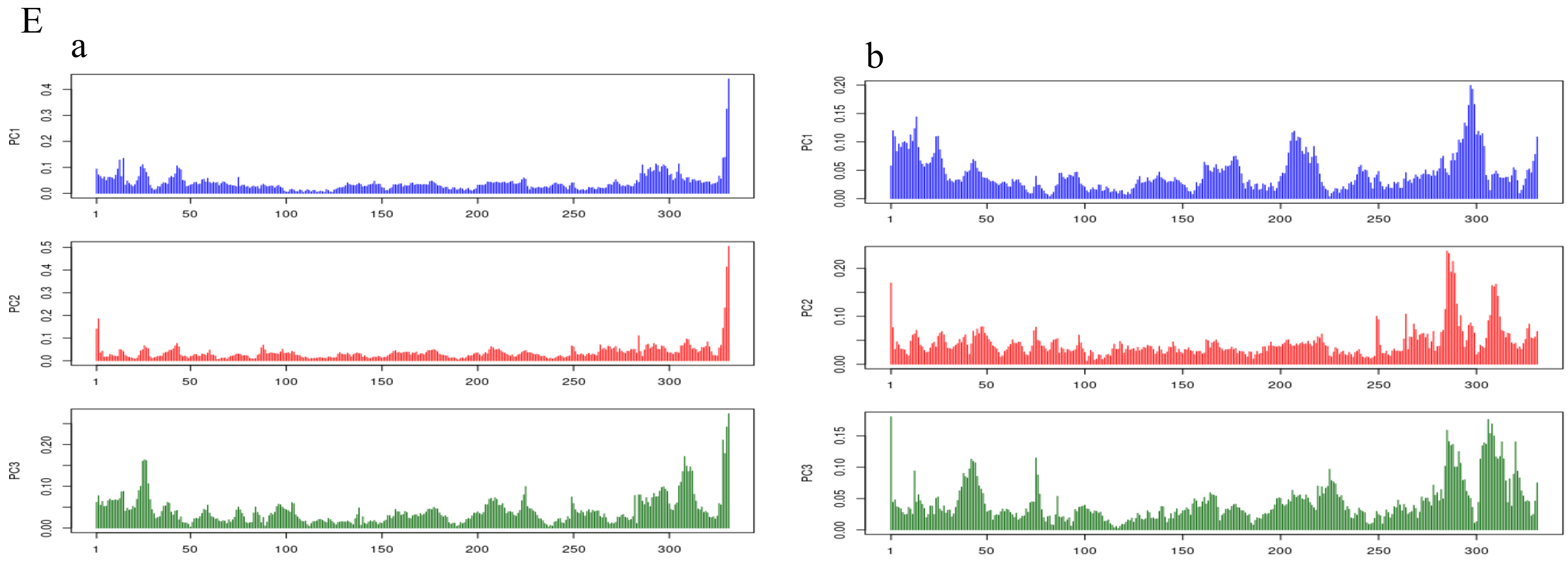

Figure S4: Variation of first three principal components (PC1, PC2 and PC3) with residues in AChE-FM (A,a), AChE-FMS (A,b), AMPK-FN (B,a), AMPK-FNS (B,b), ASK1-FM (C,a), ASK1-FMS (C,b), GST-IM (D,a), GST-6CIPHD (D,b), PKC-IM (E,a) and PKC-6CIPHD (E,b) complexes.
